# The rare entity of gastrointestinal leiomyosarcomas: An Italian multicenter retrospective study in high‐volume referral centers

**DOI:** 10.1002/cam4.6340

**Published:** 2023-07-16

**Authors:** Paola Zagami, Alessandro Comandone, Marco Fiore, Giacomo Giulio Baldi, Giovanni Grignani, Bruno Vincenzi, Alessandro Gronchi, Gabriele Antonarelli, Antonella Boglione, Elisabetta Pennacchioli, Giuseppe Curigliano, Fabio Conforti, Tommaso Martino De Pas

**Affiliations:** ^1^ Department of Oncology and Hematology University of Milan Milan Italy; ^2^ SC oncologia, Ospedale San Giovanni Bosco ASL Città di Torino Turin Italy; ^3^ Fondazione IRCCS Istituto Nazionale dei Tumori Milan Italy; ^4^ Department of Medical Oncology Santo Stefano Hospital Prato Italy; ^5^ Medical Oncology Candiolo Cancer Institute‐FPO, IRCCS Candiolo Italy; ^6^ Department of Oncology AOU Citta della Salute e della Scienza di Torino Torino Italy; ^7^ Department of Medical Oncology Università Campus Bio‐Medico di Roma Rome Italy; ^8^ Surgical Division of Melanoma and Sarcoma European Institute of Oncology, IRCCS Milan Italy; ^9^ Division of New Drugs and Early Drug Development for Innovative Therapies European Institute of Oncology, IRCCS Milan Italy; ^10^ Division of Medical Oncology of Melanoma Sarcoma and Rare tumors, European Institute of Oncology, IRCCS Milan Italy; ^11^ Oncology Department Humanitas Gavazzeni Bergamo Italy

**Keywords:** gastrointestinal leiomyosarcoma, localized disease, metastatic de novo disease, multidisciplinary, rare tumors, sarcoma, surgery, treatment

## Abstract

**Background:**

After a huge efficacy of imatinib in treating patients with gastrointestinal stromal tumors (GISTs) was proven, a maximum effort was made to make a differential diagnosis between GISTs and gastrointestinal leiomyosarcomas (GI‐LMS), showing the latter to be an extremely rare tumor entity. Limited data on GI‐LMS biology, clinical behavior and drug‐sensibility are available, and the clinical decision‐making in this subgroup of patients is usually challenging.

**Methods:**

We conducted a multicenter, retrospective observational study on patients with diagnosed GI‐LMS from 2004 to 2020 within six high‐volume referral centers in Italy.

**Results:**

Thirty‐three patients had diagnosis of KIT‐negative GI‐LMS confirmed by sarcoma‐expert pathologist. The most common site of origin was the intestine. Twenty‐two patients had localized disease and underwent surgery: with a median follow‐up of 72 months, median disease‐free survival was 42 months. Overall survival (OS)‐rate at 5 years was 73% and median OS was 193 months. Five out of 10 patients with local relapse received a salvage surgery, and 2/5 remained with no evidence of disease. Thirteen patients received neoadjuvant (6) or adjuvant (7) chemotherapy, and 2/13 patients remained free from relapse. The median OS for patients with metastatic LMS was 16.4 months.

**Conclusion:**

GI‐LMS is very rare and extremely aggressive subgroup of sarcomas with a high tendency to systemic spread. Localized GI‐LMS at diagnosis may be cured if treated with adequate surgery with or without (neo) adjuvant chemotherapy, while de‐novo metastatic disease appeared to have a poor prognosis. Clinical effort to understand GI‐LMS biology and clinical behavior and to develop active treatment strategy, especially for metastatic‐disease, is warranted.

## 1 INTRODUCTION

Leiomyosarcomas (LMS) are malignant mesenchymal tumors accounting for approximately 10%–20% of all soft tissue sarcoma (STS).^1^ They originate from mesenchymal cells, and may arise potentially from all organs of the body, including intestines, stomach, bladder, blood vessels (predominantly veins) and, most frequently, the uterus or soft tissue. Compared to other sarcoma types, LMS less commonly arises from the extremities.^2^ In general, the overall incidence of LMS increases with age, reaching its peak at the seventh decade of life.^3^ Although there are no clear predisposing factors to the development of LMS, radiation exposure as well as genetic syndromes, such as Li‐Fraumeni and hereditary retinoblastoma, have been associated with an increased risk for any STS, including LMS.^4^, ^5^, ^6^

Gastrointestinal (GI) LMS represents a rare subgroup of LMS, and most commonly derives from the colorectum (40%), small intestine (40%), stomach or esophagus (10% each).^7^ Until 2000, gastrointestinal stromal tumors (GISTs) had often been misdiagnosed as GI‐LMS, from which they differ both in terms of prognosis as well as treatment modalities. In 1998 after Hirota et al. seminal paper about the mutation in the KIT gene, characterizing GIST, it became easy to diagnose these two types of GI STS, based on CD117 (KIT) and CD34 along with smooth muscle actin or desmin immunostaining.^8^, ^9^, ^10^ Since then, a better understanding of the incidence and distribution of GI‐LMS became evident.^11^ While GIST are the commonest sarcoma in general and the commonest sarcoma of the GI tract, GI‐LMS are ultrarare. Diagnosis of GIST needs the identification of activating mutations in KIT or PDGFRA (molecular alterations) and expression of CD117 and/or CD34 (immunohistochemistry); while GI‐LMS express smooth muscle actin and desmin. The prognosis and treatment of these two GI‐ sarcoma are very different. GIST have a better prognosis than GI‐LMS thanks also to the very effective target therapies. For localized GIST, surgery is the mainstream treatment following to adjuvant target therapy based on the risk. For metastatic GIST, KIT‐inhibitors (e.g., imatinib or avapritinib) (anti‐KIT/PDGFRA) or other anti‐angiogenic (e.g. sunitinib) agents are available and effective to control the disease.

The treatment for GI‐LMS is derived from the standard of care for other originating in other sites.^12^ It includes radical surgery for localized disease, systemic treatment in case of metastatic spreading, in the absence of data on the effectiveness of perioperative chemotherapy.^12^ Due to the extreme rarity of GI‐LMS, very few data are available, mainly derived from retrospective analyses and case report.^13^, ^14^, ^15^, ^16^

Based on these limited data, and considering the rarity of this subgroup of sarcomas and the big unmet need to understand its biology and clinical behavior, the aim of our study is to describe the clinical characteristics, as well as the long term outcomes of a cohort of patient with GI‐LMS treated within high‐volume referral centers in Italy.

## 2 METHODS AND ANALYSIS

We conducted a multicenter, retrospective study on patients with diagnosis of GI‐LMS between 2004 and 2020 treated at six high‐volume referral centers in Italy. All patients had a histologically confirmed diagnosis of GI‐LMS by sarcoma‐expert pathologist valuing morphological characteristics, as well as exclusion of KIT expression on immunohistochemistry (IHC). Tumors originating from esophagus, stomach, small intestine, colon or anal rectum were included in this analysis. We retrospectively reviewed electronic patient records to extract demographic, clinical, therapy‐related, and follow‐up data. All patients were treated following a multidisciplinary treatment indication. A chest, abdomen, pelvis computed tomography scan was performed every 4–6 months for the first 2 years in patients with localized disease who underwent surgery, while patients who received palliative systemic chemotherapy had a restaging CT‐scan every 2–4 months. Descriptive analyses were performed for all patients.

Disease‐free survival (DFS), distant metastasis–free survival (DMFS) and overall survival (OS) functions were estimated using the Kaplan–Meier method on the numbers of patients with available survival data.

Disease‐free survival was calculated from the date of surgery to the date of invasive relapse (categorized as loco‐regional events, and distant metastases), appearance of a second primary cancer, or death, whichever occurred first. OS was calculated from date of diagnosis to date of death or last follow‐up.

The cumulative incidence of distant metastases (CI‐DM) curve function was estimated according to methods described by Kalbfleisch and Prentice, taking into account the competing causes of recurrence.

All statistical analyses were performed using R software. The study, named IEO1618, was approved by the Institutional Review Board and patients signed adequate document for informed consent.

## 3 RESULTS

**FIGURE 1:**
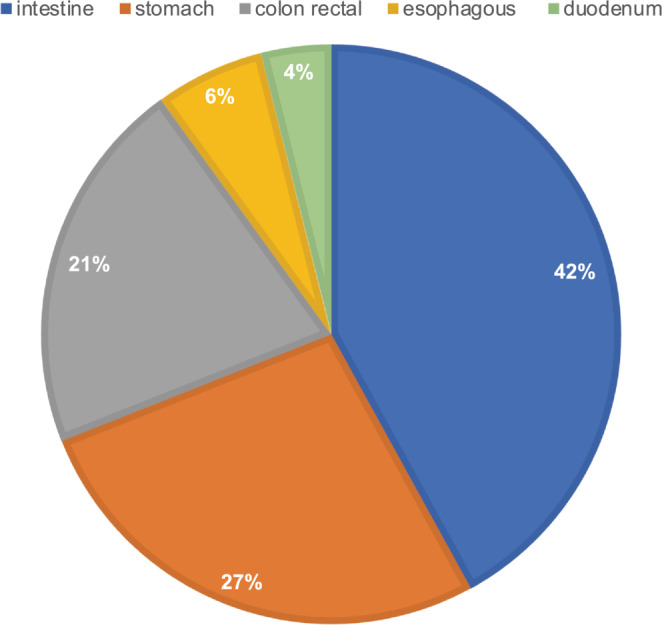
Distribution of GI LMS by organ of origin.

Between 2004 and 2020, 33 patients with GI‐LMS were referred and treated in the participating institutions. Clinical‐pathological characteristics are described in Table 1. Median age at diagnosis was 57 years. The most common site of origin was the small bowel, followed by the stomach and colon‐rectum (Figure 1). Most of the population had localized disease (22/33) with fewer cases of de novo metastatic diagnosis (11/33), mostly with liver and peritoneum involvement. The most common presenting symptom of GI‐LMS was abdominal pain. More than 90% (30/33) of patients, of whom 21 and 9 with localized and metastatic disease at diagnosis respectively, received surgical treatment, and 19 out of 30 (70%) resulted macroscopically complete. Six patients received neoadjuvant anthracycline‐based chemotherapy. Specifically, two combined with dacarbazine, two with ifosfamide, one in combination with both. Two of these patients did not manage to undergo to surgery for rapid local and abdominal disease progression. No patient received neoadjuvant or adjuvant radiotherapy, while seven patients received adjuvant chemotherapy. There were no significant differences in clinicopathologic‐characteristics between patients with local relapse or not as well as with distant relapse (Tables 2 and 3).

**TABLE 1:** Clinic‐characteristics of patients.

Characteristic	*N* = 33
Sex	
Female	15 (45%)
Male	18 (55%)
Stage at diagnosis	
Localized	22 (67%)
Metastatic	11 (33%)
Site of metastases at diagnosis	
Liver	7 (64%)
Peritoneum	4 (36%)
Unknown	22
Size of primary tumor (cm)	
Unknown	8 (24%)
< 5	6 (18%)
> 5	19 (58%)
Grade of primary tumor	
3	16 (62%)
2	6 (23%)
1	4 (15%)
Unknown	7
Site of origin of primary tumor	
Colon‐rectal	7 (21%)
Duodenum	1 (3.0%)
Esophageal	2 (6.1%)
Intestine	14 (42%)
Stomach	9 (27%)
Surgery	
Yes	30 (91%)
No	3 (9.1%)
Outcome of surgery	
Radical	19 (70%)
marginal	8 (30%)
Unknown	6
Adjuvant chemotherapy	
Yes	7 (24%)
No	22 (76%)
Unknown	4
Neoadjuvant chemotherapy	
Yes	6 (19%)
No	26 (81%)
Unknown	1
Local relapse	
Yes	10 (33%)
No	20 (67%)
Unknown	3
Distant relapse	
Yes	20 (65%)
No	11 (35%)
Unknown	2
Site of distant relapse	
Liver	14 (74%)
Liver and lung	1 (5.3%)
Periitoneum	3 (16%)
Retroperitoneum	1 (5.3%)
Unknown	14

*Note*: Values are expressed as *n* (%).

**TABLE 2:** Characteristics of the patients with local relapse.

Characteristic	Local relapse = 10	Non local relapse = 20	*p*‐Value^a^
Sex			0.7
Female	4 (40%)	10 (50%)	
Male	6 (60%)	10 (50%)	
Dimension of primary tumor (cm)			0.5
Unknown	3 (30%)	3 (15%)	
< 5	1 (10%)	5 (25%)	
> 5	6 (60%)	12 (60%)	
Grade of primary tumor			0.2
3	8 (80%)	5 (38%)	
2	1 (10%)	5 (38%)	
1	1 (10%)	3 (23%)	
Unknown	0	7	
Site of primary tumor			0.4
Colon‐rectal	1 (10%)	6 (30%)	
Duodenum	1 (10%)	0 (0%)	
Esophageal	1 (10%)	1 (5.0%)	
Intestine	5 (50%)	7 (35%)	
Stomach	2 (20%)	6 (30%)	
Surgery			0.3
Yes	8 (80%)	19 (95%)	
No	2 (20%)	1 (5.0%)	
Outcome of surgery			0.3
Radical	4 (57%)	14 (82%)	
Marginal	3 (43%)	3 (18%)	
Unknown	3	3	
Adjuvant chemotherapy			>0.9
Yes	2 (25%) (EPI + IFO)	4 (21%) (DTIC; DTIC+ADM; GEM; TAX)	
No	6 (75%)	15 (79%)	
Unknown	2	1	
Neoadjuvant chemotherapy			0.14
Yes	4 (40%) (MAID; DTIC+ADM; ADM; EPI + IFO)	2 (11%) (DTIC+ADM;EPI + IFO)	
No	6 (60%)	17 (89%)	
Unknown	0	1	
Distant relapse			>0.9
Yes	6 (60%)	12 (63%)	
No	4 (40%)	7 (37%)	
Unknown	0	1	
Site of distant relapse			0.3
Liver	4 (80%)	8 (67%)	
Liver and lung	0 (0%)	1 (8.3%)	
Periitoneum	0 (0%)	3 (25%)	
Retroperitoneum	1 (20%)	0 (0%)	
Unknown	5	8	

*Note*: Values are expressed as *n* (%).

Abbreviations: DTIC, dacarbazine; DTIC+ADM, dacarbazine plus adriamycin; GEM, gemcitabine; IFO, ifosfamide; MAID, mesna, adriamycin, ifosfamide, dacarbazine; TAX: taxol.

^*a*^ Fisher's exact test.

**TABLE 3:** Characteristics of the patients with distant relapse.

Characteristic	Distant relapse, *N* = 20	No distant, *N* = 11	*p*‐Value^a^
Sex			0.2
Female	8 (40%)	7 (64%)	
Male	12 (60%)	4 (36%)	
Stage at diagnosis (AJCC 8th edition)			0.002
1	9 (45%)	11 (100%)	
2	11 (55%)	0 (0%)	
Dimension primary tumor (cm)			0.11
Unknown	5 (25%)	2 (18%)	
< 5	1 (5.0%)	4 (36%)	
> 5	14 (70%)	5 (45%)	
Grade of primary tumor			0.4
3	10 (71%)	5 (45%)	
2	2 (14%)	4 (36%)	
1	2 (14%)	2 (18%)	
Unknown	6	0	
Site of primary tumor			0.3
Colon‐rectal	3 (15%)	3 (27%)	
Duodenum	1 (5.0%)	0 (0%)	
Esophageal	1 (5.0%)	1 (9.1%)	
Intestine	7 (35%)	6 (55%)	
Stomach	8 (40%)	1 (9.1%)	
Surgery			>0.9
Yes	18 (90%)	10 (91%)	
No	2 (10%)	1 (9.1%)	
Outcome of surgery			0.7
Radical	10 (62%)	7 (78%)	
Marginal	6 (38%)	2 (22%)	
Unknown	4	2	
Adjuvant chemotherapy			0.7
1	5 (29%)	2 (20%)	
2	12 (71%)	8 (80%)	
Unknown	3	1	
Neoadjuvant chemotherapy			>0.9
1	4 (21%)	2 (18%)	
2	15 (79%)	9 (82%)	
Unknown	1	0	
Site of distant relapse			>0.9
Liver	14 (74%)	0 (NA%)	
Liver and lung	1 (5.3%)	0 (NA%)	
Periitoneum	3 (16%)	0 (NA%)	
Retroperitoneum	1 (5.3%)	0 (NA%)	
Unknown	1	11	

*Note*: Values are expressed as *n* (%).

^*a*^ Pearson's chi‐squared test; Fisher's exact test.

**FIGURE 2:**
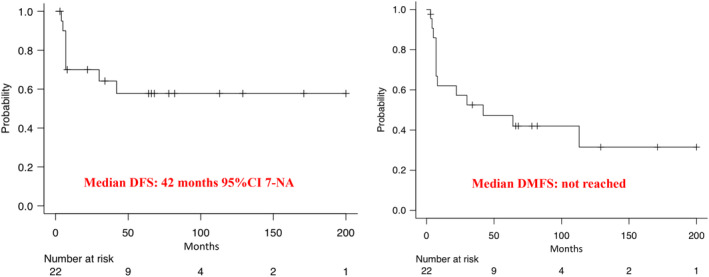
Kaplan–Meier curves of disease free survival (DFS) and distant metastasis free survival (DMFS) in localized disease at diagnosis.

**FIGURE 3:**
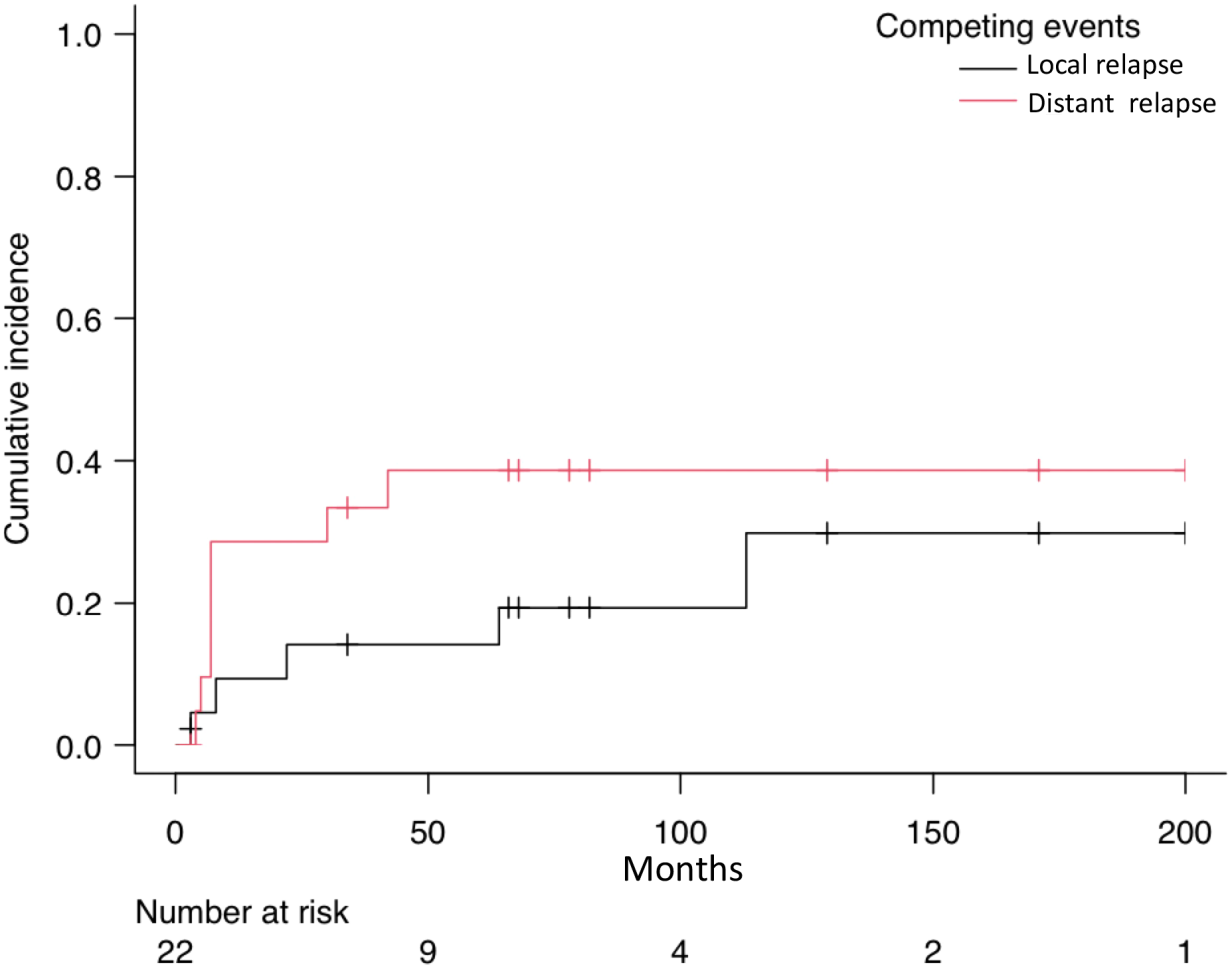
Cumulative incidence of events in localized GI‐LMS.

The median follow‐up of patients with localized disease at diagnosis (*n* = 22) was 72 months. The median disease‐free survival (mDFS) and distant metastasis–free survival (mDMFS) were of 42 months (95% CI, 7‐NA) and not reached, respectively (Figure 2). The cumulative incidence of locoregional recurrences and distant metastases at 5 years, were 14% (95% CI, 3%–32%) and 39% (95%CI, 18%–59%), respectively (Figure 3).

**FIGURE 4:**
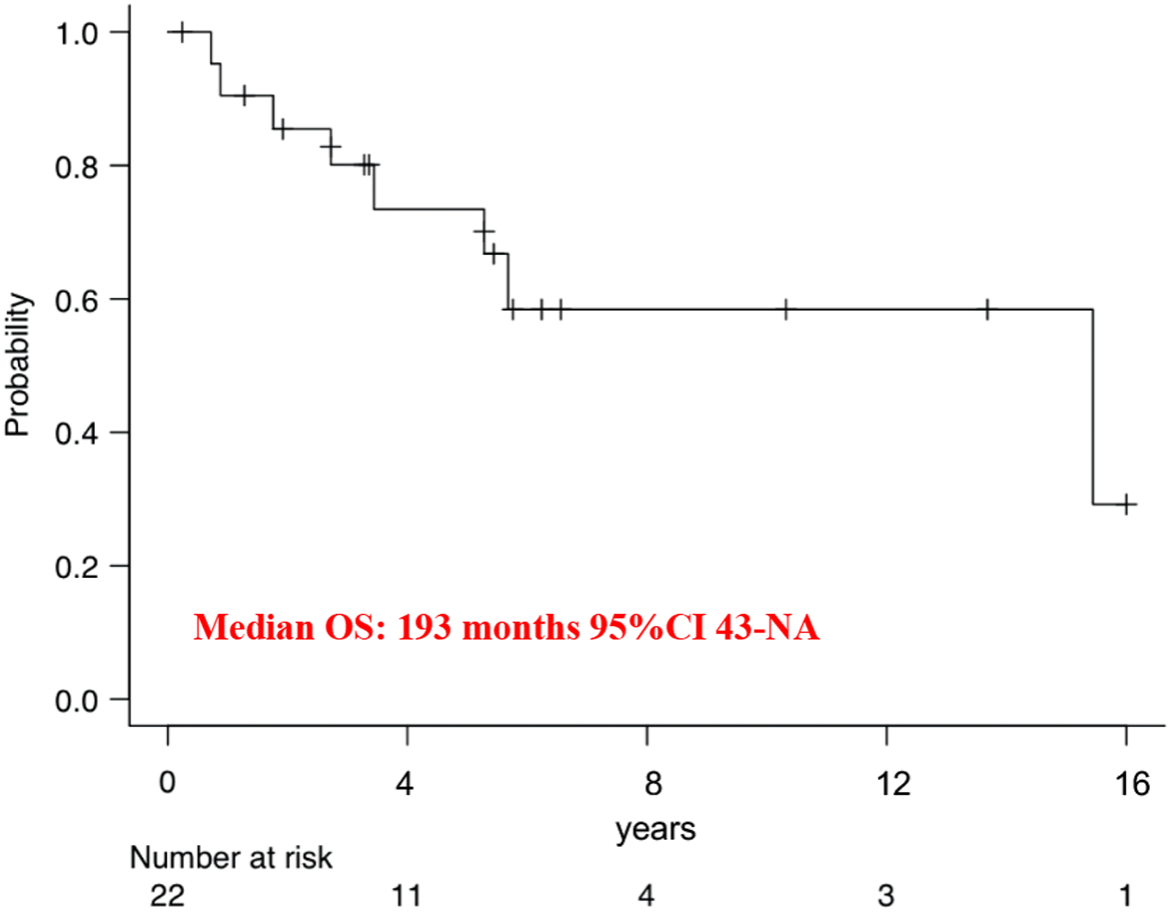
Kaplan–Meier curves of overall survival of patients with localized disease at diagnosis. CI, confidence interval; OS, overall survival.

The OS‐rate at 5 years was 73% (95% CI 47%–88%) and the median OS was 193 months (95% CI 43‐NA; Figure 4).

Five out of ten patients with local relapse after first surgery, underwent to subsequent salvage surgery. Two out of such five patients remained with no evidence of disease (NED). Out of 13 patients who received perioperative chemotherapy, 2/13 patients remained without tumor relapse.

**FIGURE 5:**
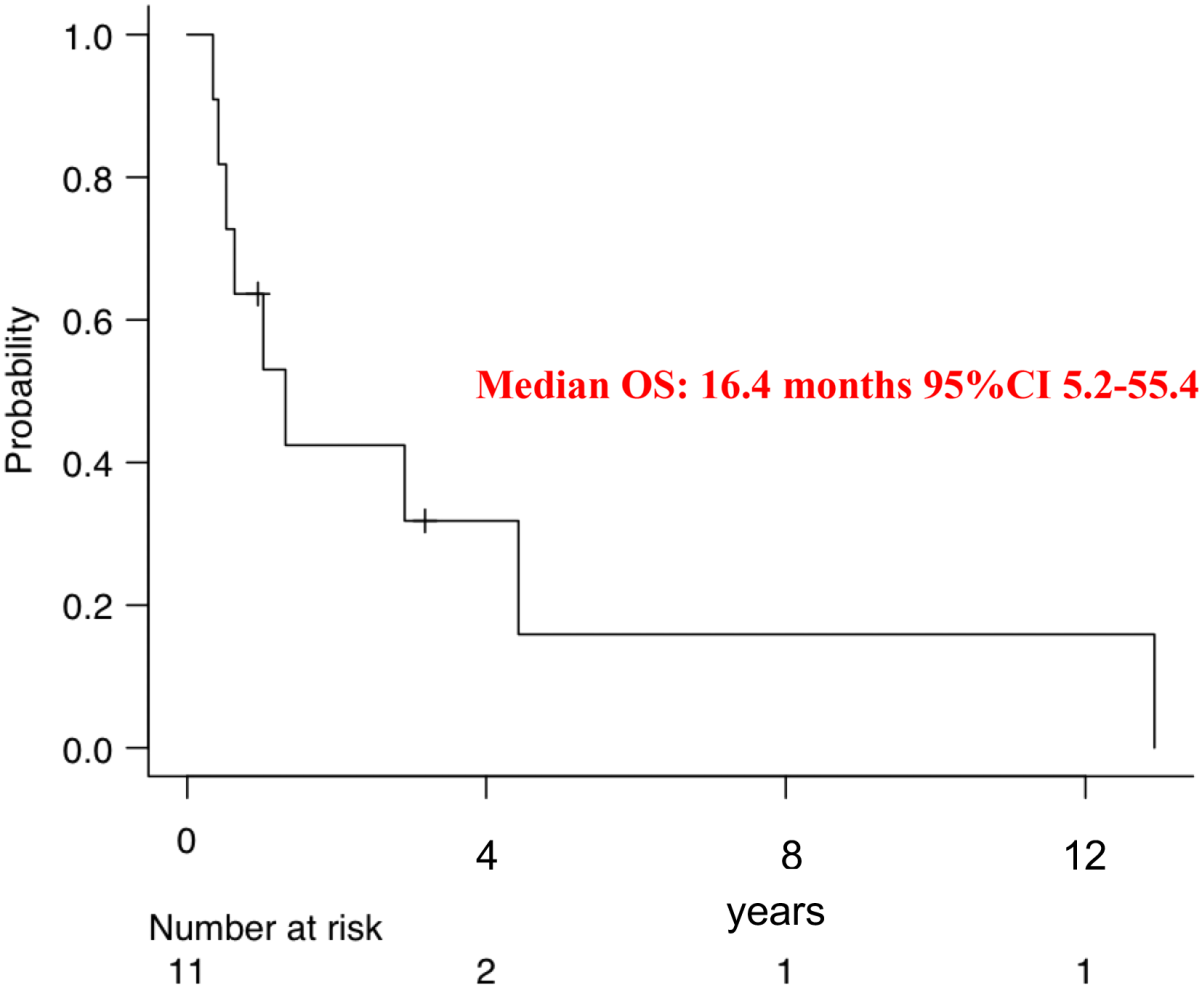
Kaplan–Meier curves of overall survival of patients with metastatic disease at diagnosis. CI, confidence interval; OS, overall survival.

Overall, patients with advanced disease at diagnosis (*n* = 11) had poor prognosis, with a median OS of only 16.4 months (95% CI 5.2–55.4; Figure 5) and 3‐ and 5‐year OS‐rate of 42% (95% CI, 14–69) and 16% (95% CI 1%–47%), respectively. Nine patients received surgery on primary tumor and three had local relapse; one after radical surgery, one after marginal surgery and one showed rapidly local and systemic progression.

## 4 DISCUSSION

This is an observational study reporting the epidemiology and the clinical features of a retrospective cohort of patients diagnosed with GI LMS from 2004 to 2020 treated within six high‐volume referral centers in Italy. All the diagnoses were confirmed by a sarcoma‐expert pathologists. Only tumors with absence of KIT (and DOG1 since routinely used)‐expression were included, so as to exclude from the population included in the study patients with GISTs.

In line with the literature, the small number of cases collected among six referral centers confirms that GI‐LMS represent a very‐rare entity of STS.

Our data showed that patients with localized disease treated in referral centers may be cured if treated with surgery with or without (neo) adjuvant chemotherapy. Patients with localized disease at diagnosis had a median DFS of 42 months, with more than half of patients without distant metastases after 5 years from surgery. This observation supports an effort for early diagnosis and macroscopically complete resection whenever possible.

Our data also suggest that when local relapse occurs, an effort for a salvage surgery should be done. In our series, two out of five patients who underwent salvage surgery for local relapse remained with NED.

Due to the extreme rarity of GI LMS, very few data are available in literature, mainly deriving from retrospective analyses limiting the comparison with other evidence. However, in our study the median DFS of patients with localized disease was longer than what observed among 46 GI‐LMS with high risk of recurrence even after complete resection by Smrke et al.^17^ Noteworthy, only 11 out of these 36 localized tumors were resected in sarcoma‐specialized surgery department.^17^ This difference could be related to the distinct radical surgery rate and management of the disease. In our series more than 60% of patients had localized disease at diagnosis and more than 90% received surgical treatment, of whom 70% resulted macroscopically completed. Radical surgery as the mainstay of treatment was already reported by Hilal et al in a large retrospective GI LMS series supporting our results.^18^

Albeit limited numbers precluded definitive conclusions, the better outcome observed in our cohort of patients with localized disease when compared with the few published available data supports the importance of managing this rare subgroup of patients by reference oncological centers for rare tumors, in order to improve outcomes of cancer patients, as already demonstrated in large series.^19^, ^20^

There is too little data on the role of perioperative chemotherapy to draw definitive conclusions. In previous published data conventional chemotherapy (usually anthracycline‐based therapy combined with dacarbazine or ifosfamide like in LMS of other sites of origin) achieved a median progression free survival between 2 and 8 months with no conclusion of consistent benefit from (neo)adjuvant systemic treatment due to low number of treated patients.^17^, ^18^ Of note, in our experience, only 2/13 patients (receiving dacarbazine and adriamycin plus ifosfamide) who received perioperative chemotherapy remained without tumor relapse.

In a cohort of 523 patients diagnosed with GI‐LMS from the Surveillance, Epidemiology and End Results (SEER) database, GI‐LMS with gastric or esophagus primary site were more frequently diagnosed at stage IV and the presence of distant metastases and grade 3 were associated with worse survival in both univariate and multivariate analysis.^21^ Our results showed more than 60% of the patients had localized disease at diagnosis but included a low percentage of gastric and esophageal LMS. In line with our series, the OS rate was higher in patients who underwent to surgery than who did not as.^21^ It is noteworthy that these analyses used a national database, not selecting data from sarcoma‐referral center, and carries inherent limitations resulting.^21^

Finally, the prognosis of patients with de‐novo metastatic disease in our series appeared very dismal and is consistent with previous data showing that patients with de novo metastatic disease displayed a shorter OS than those with distant recurrence (19 vs. 27 months).^17^ This observation underlies the very poor prognosis of this group of patients, suggesting a limited efficacy of systemic treatments used in soft tissue LMS sarcomas in this subset of patients and emphasizing the need to investigate new therapeutic strategies.

The strength of this study is the collection of data about an ultrarare soft tissue tumors with very limited published evidence. Moreover, all the histological diagnosis were confirmed by sarcoma‐expert pathologists, taking part of the recognized referred pathologist centers for rare tumors. The management of all collected GI LMS derived from high‐volume sarcoma referral centers as recommended by the international guideline for sarcoma. Long follow up with available data, supported the collection of adequate and trusted numbers of events for long‐term outcomes analysis.

The limitation of this analysis is the retrospective nature of the study and the heterogeneity of the population and the limited number of the patients easily explained by the ultrarare entity of this sarcoma.

Additional data are needed to enlarge the knowledge of biology and clinical behavior of GI LMS, to dissect the potential role of (neo)adjuvant treatment in order to develop multidisciplinary treatment strategy, and to improve the efficacy of systemic therapy for patients with advanced disease.

## AUTHOR CONTRIBUTIONS

**Paola Zagami:** Conceptualization (equal); data curation (lead); formal analysis (supporting); methodology (supporting); resources (supporting); supervision (equal); validation (equal); visualization (equal); writing – original draft (lead); writing – review and editing (lead). **Alessandro Comandone:** Data curation (equal); investigation (equal); resources (equal); validation (equal); visualization (equal); writing – review and editing (equal). **Marco Fiore:** Data curation (equal); investigation (equal); resources (equal); validation (equal); visualization (equal); writing – review and editing (equal). **Giacomo Giulio Baldi:** Data curation (equal); investigation (equal); resources (equal); validation (equal); visualization (equal); writing – review and editing (equal). **Giovanni Grignani:** Data curation (equal); investigation (equal); resources (equal); validation (equal); visualization (equal); writing – review and editing (equal). **Bruno Vincenzi:** Data curation (equal); investigation (equal); resources (equal); validation (equal); visualization (equal); writing – review and editing (equal). **Alessandro Gronchi:** Data curation (equal); investigation (equal); resources (equal); validation (equal); visualization (equal); writing – review and editing (equal). **Gabriele Antonarelli:** Validation (equal); visualization (equal); writing – original draft (supporting); writing – review and editing (equal). **Antonella Boglione:** Data curation (equal); investigation (equal); resources (equal); validation (equal); visualization (equal); writing – review and editing (equal). **Elisabetta Pennacchioli:** Data curation (equal); investigation (equal); resources (equal); validation (equal); visualization (equal); writing – review and editing (equal). **Giuseppe Curigliano:** Supervision (equal); validation (equal); writing – review and editing (equal). **Fabio Conforti:** Conceptualization (equal); data curation (equal); formal analysis (lead); investigation (equal); methodology (lead); resources (equal); supervision (equal); validation (equal); visualization (equal); writing – original draft (equal); writing – review and editing (equal). **Tommaso Martino De Pas:** Conceptualization (equal); data curation (equal); investigation (lead); project administration (lead); resources (equal); supervision (lead); validation (equal); visualization (equal); writing – original draft (equal); writing – review and editing (equal).

## FUNDING INFORMATION

This research did not receive any specific grant from funding agencies in the public, commercial, or not‐for‐profit sectors.

## CONFLICT OF INTEREST STATEMENT

The authors declare that they have no known competing financial interests or personal relationships that could have appeared to influence the work reported in this article.

## Data Availability

Data sharing is not applicable to this article as no new data were created or analyzed in this study.
